# Low-dose anticoagulation after isolated mechanical aortic valve replacement with Liva Nova Bicarbon prosthesis: A post hoc analysis of LOWERING-IT Trial

**DOI:** 10.1038/s41598-018-26528-9

**Published:** 2018-05-30

**Authors:** Michele Torella, Iolanda Aquila, Paolo Chiodini, Cristiano Amarelli, Gianpaolo Romano, Ester Elena Della Ratta, Marisa De Feo, Alessandro Della Corte, Ciro Indolfi, Daniele Torella, Luca Salvatore De Santo

**Affiliations:** 1 Department of Cardio-Thoracic Sciences, University of Campania Luigi Vanvitelli, Naples, Italy; 2 Cardiovascular Institute, Department of Medical and Surgical Sciences, Magna Graecia University, Catanzaro, Italy; 3 Department of Medicine and Public Health, University of Campania “Luigi Vanvitelli”, Naples, Italy; 4 Department of Cardiovascular Surgery and Transplants, V. Monaldi Hospital, Naples, Italy; 5 Department of Medical and Surgical Sciences, Chair of Cardiac Surgery, University of Foggia, Foggia, Italy; 6 Casa di Cura Montevergine, GVM, Care & Research, Avellino, Italy

## Abstract

Thromboembolic complications after cardiac valve replacement are due to a complex interplay between patients’ characteristics, device features and anticoagulation intensity. Subtle design and material differences in available prostheses may thrombosis. We conducted a post-hoc sub-analysis of the LOWERING-IT database to test the safety and feasibility of a low-level oral anticoagulant regime in low-risk patients with aortic LivaNova prosthetic valve replacement. The study population included 148 patients randomized to a low INR target (1.5–2.5; LOW-INR group), and 144 patients to the standard INR (2.0–3.0; CONVENTIONAL-INR group). The non-inferiority of thromboembolic events between LOW-INR and CONVENTIONAL-INR groups was tested. Cumulative follow-up reached 1,545 patient/years. The mean INR was 1.91 ± 0.23 in the LOW-INR group, and 2.59 ± 0.26 in the CONVENTIONAL-INR group (P < 0.001). There were 3 thromboembolic events, all in the CONVENTIONAL-INR group. Comparison of thromboembolic events was not significant. The 1-sided 97.5% exact CI for the difference in primary event proportion was 0.54%, satisfying criteria non-inferiority. Bleeding events were significantly different: 6.61 per 1,000 patient-year in LOW-INR group vs 18.65 per 1,000 patient-year in CONVENTIONAL-INR group (p < 0.045, RR 0.37). In conclusions these data suggest that low-dose anticoagulation is safe in selected patients after aortic LivaNova Bicarbon prosthesis implantation.

## Introduction

Despite significant improvements, mechanical prosthetic heart valve implantation is still veiled by the inherent risk of thrombosis and systemic embolism. The burden of these complications is significantly reduced by lifelong oral anticoagulant therapy with vitamin K antagonist (VKA) therapy, which is however associated with an increased risk of bleeding. Current knowledge is that the risk of developing thromboembolism and bleeding depends on a complex interplay between individual patient’s characteristics, the type of prosthetic valve, optimal VKA dose scheduling and the intensity of anticoagulation^1–4^.

The optimal intensity of anticoagulant therapy, defined as the level at which the incidence of both thromboembolic and bleeding complications is lowest, remains a delicate equilibrium and continues to be matter of debate. A lower level of anticoagulation will allow a decreased risk of hemorrhage, but its efficacy on thromboembolic risk is less defined^5^.

The LOWERING-IT was an open-label, randomized controlled trial carried out to test the hypothesis that a low intensity level of oral anticoagulant regime with an international normalized ratio (INR) range between 1.5 to 2.5 (LOW-INR) is as effective and safe as a higher level of anticoagulant therapy with a recommended (standard) INR range of 2.0 to 3.0, in low-risk patients with a single aortic mechanical prosthetic valve replacement. The hypothesized outcome from using a 1.5 to 2.5 INR intensity level (as opposed to the currently recommended INR of 2.0 to 3.0) was a reduction in the incidence of hemorrhagic episodes without affecting the risk of thromboembolic events. LOWERING-IT trial established that the proposed LOW-INR target is safe and feasible in low-risk patients after bileaflet aortic mechanical valve replacement. It results in similar thrombotic events incidence and significant reduction of bleeding occurrence when compared to conventional anticoagulation regimen^6^. The data from LOWERING-IT trial were afterwards revised and included in the current guidelines on oral anticoagulation for patients with isolated aortic valve disease undergoing single bileaflet mechanical aortic valve replacement (AVR)^7,8^.

An intriguing and, to some extent, confounding aspect of the LOWERING-IT trial is that the study population consisted of patients receiving different types of prosthetic valves with most of them implanted with a LivaNova Bicarbon bileaflet mechanical aortic valve. The latter data inferred that there might be a potential beneficial effect of the low intensity level oral anticoagulant regime for patients receiving the LivaNova Bicarbon valves, because of their distinguished details in materials and design.

Therefore, we conducted a post hoc analysis of the LOWERING-IT trial database to test the safety and feasibility of a low-level oral anticoagulant regime with an INR range between 1.5 and 2.5 compared to a higher level of anticoagulant therapy with the recommended INR range of 2.0 to 3.0 in low-risk patients with aortic LivaNova Bicarbon prosthetic valve replacement.

## Methods

### Study design and anticoagulant therapy

The LOWERING-IT study was a prospective, open-label, single-center randomized controlled trial that compared the thromboembolic and bleeding events between 2 different anticoagulation intensity levels in low-risk patients undergoing a single aortic mechanical prosthetic valve replacement. The 2 anticoagulation intensity levels were the low anticoagulation intensity, with an INR range of 1.5 to 2.5 (LOW-INR group), and the currently recommended intensity, with the standard INR range of 2.0 to 3.0 (CONVENTIONAL-INR group). Anticoagulation levels were achieved with vitamin K antagonists - VKAs (Warfarin) after the mechanical prosthetic AVR. Of note, no aspirin was added.

The research protocol was approved by Monaldi Hospital Ethical Committee, and the study was carried out in accordance with the Declaration of Helsinki. The patients gave informed consent before surgery.

The postoperative anticoagulant treatment was standardized with low-molecular-weight heparin alone, which began 6 hours after the end of cardiopulmonary bypass and was then administered until the target INR level was reached. The low-molecular-weight heparin regimen used after surgery as a “bridge” to warfarin consisted of henoxaparin sodium 4,000 IU every 12 hours as per internal protocol used in our institution for low-risk patients. Oral anticoagulant therapy started after surgical drainage removal. Warfarin (Coumadin, Bristol-Myers Squibb) was administered to maintain the INR within the assigned range.

The follow-up was conducted by the investigators in person. The investigators carrying out the routine INR evaluation were responsible for the warfarin dose adjustment and frequency of INR testing, which was roughly assessed every 3 weeks at our institution. All INR test results were compiled from the time of randomization to the end-of-study visit. Any bleeding events were reported by the patients and adjudicated by the investigators if present at the follow-up visits or by a thorough clinical anamnesis investigation of the period preceding the checkup visit. Thrombotic and main bleeding events were adjudicated at hospital admittance. Finally, patients underwent complete clinical checkup visits (including electrocardiogram, echocardiography, and electrocardiogram-Holter) at 1, 3, 6, and 12 months during the first year after surgery and every 6 months thereafter.

The trial is registered with controlled-trials.com (ISRCTN34082835 - http://www.controlled-trials.com/ISRCTN34082835).

### Study end points

The primary outcome was the thromboembolic events. These included valve thrombosis, ischemic stroke, Transient ischemic attack (TIA), and coronary and/or peripheral embolism.

Major secondary outcome was the occurrence of bleeding events. These included intracranial and spinal bleeding, major extracranial bleeding, and minor extracranial bleeding. Hemorrhagic events were considered to be major when blood transfusion, hospitalization, or a surgical procedure was required.

Other secondary outcomes were the occurrence of endocarditis, atrial fibrillation, withdrawal from the oral anticoagulant therapy, and death.

### Sample size

The LOWERING-IT trial was designed to assess the noninferiority of thromboembolic events between LOW-INR and CONVENTIONAL-INR groups. In particular, in the LOWERING-IT trial 148 patients with LivaNova Bicarbon prosthesis were randomized to LOW-INR group, while 144 patients with LivaNova Bicarbon prosthesis were randomized to CONVENTIONAL-INR group. In this report a subgroup analysis was performed including only these patients. The subgroup analysis achieved a 70% power, at a significance level of 0.025 (1-sided) and considering the assumption of the LOWERING-IT trial. Specifically, an expected 4-year proportion in the standard CONVENTIONAL-INR group of 0.016 and a noninferiority margin delta of 0.036 (corresponding to 0.90%/year).

### Statistical analysis

Continuous variables were expressed as mean and SD, whereas categorical variables were expressed as absolute number and percentage. The proportion of the intention-to-treat population experiencing primary events for both treatment groups and the associated 1-sided 97.5% exact CI for the difference were estimated. The noninferiority margin (Δ) defined in the primary analysis was based on absolute event proportion differences. Noninferiority of LOW-INR over CONVENTIONAL-INR target was accepted if the upper bound of the 97.5% exact CI around the estimated difference in primary event proportion lies below Δ. For this subgroup analysis, the same absolute Δ of 3.6% at 4 years of the LOWERING-IT trial was adopted. Fisher exact test and Relative Risk (RR) with exact 95% CI were used for the comparison of primary outcome variable between the two groups. Comparison of hemorrhagic events between the two groups was performed using asymptotic Pearson Chi-square test and RR including 95% CI. The linearized incidence rate was determined by dividing the number of events by the total number of person-years accumulated. The 95% CI around the estimates were calculated based on the Poisson distribution. Statistical analyses were performed using SAS version 9.4 (SAS Institute, Cary, NC). Exact tests were performed using StatXact 9 (Cytel Software, Cambridge, MA).

### Disclosure

The authors received an unrestricted grant from LivaNova to perform this post-hoc analysis.

## Results

### Baseline characteristics and follow-up

A total of 292 patients were included in this subgroup analysis (148 patients in LOW-INR group and 144 patients in CONVENTIONAL-INR group).

The baseline characteristics and the surgical data of the patients included in this report are shown in Tables 1 and 2. The 2 groups were well matched with respect to baseline characteristics and procedural characteristics. Accordingly, the patient characteristics of the study group are comparable to those of the general LOWERIN-IT population^6^.

**Table 1:** Baseline characteristics of the patients.

	LOW-INR Group, n. (%)	CONVENTIONAL-INR Group, n. (%)
No. of patients	148	144
Age, year mean (SD)	50.2 (7.9)	49.9 (8.7)
Sex male	104 (70.3%)	112 (77.8%)
Hypertension	77 (52.0%)	68 (47.2%)
BMI, kg/m^2^ mean (SD)	27.50 (4.28)	27.52 (4.45)
NYHA class		
I	42 (28.4%)	11 (7.6%)
II	87 (58.8%)	102 (70.8%)
III–IV	19 (12.8%)	31 (21.5%)
Valve disease		
Regurgitation	69 (46.6%)	77 (53.5%)
Stenosis	41 (27.7%)	18 (12.5%)
Mixed	38 (25.7%)	49 (34.0%)

**Table 2:** Surgical data.

	LOW-INR Group, n. (%)	CONVENTIONAL-INR Group, n. (%)
Extracorporeal time, min	88.8 (32.9)	83.3 (23.7)
Aortic clamp time, min	61.4 (19.4)	58.8 (16.9)
Type of prosthesis		
LivaNova Bicarbon	148 (75.1%)	144 (72.4%)
Valve diameter		
21 mm	38 (25.7%)	33 (22.9%)
23 mm	80 (54.1%)	76 (52.8%)
>23 mm	30 (20.3%)	35 (24.3%)

The patients were followed up for a total 1,545 patient-years (1,545 patient-years; 757 patient-years in the CONVENTIONAL-INR group and 788 patient- years in the LOW-INR group). The median study follow-up was 5.3 years (range 1.2–7.4). For all 292 patients, a total of 34,576 measurements of the INR were obtained during the follow-up period, with a median of 17 (range 15–24) measurements per year for each patient. The mean INR in the LOW-INR group was 1.91 ± 0.23, whereas in the CONVENTIONAL-INR group, it was 2.59 ± 0.26 (P < 0.001).

Eighty-six percent of the INR values measured in the LOW-INR group and 84% of the INR values measured in the CONVENTIONALINR group were within the group’s target range. These values remained stable in both outpatient groups throughout the study period. All patients remained in the assigned target group during follow-up.

### Outcome events

Table 3 summarizes the number of patients who experienced at least one critical event. During the entire study period, 21 complications occurred. Thrombotic and major bleeding events (Table 4) occurred all during the first 24 months of follow-up, whereas minor bleeding events were spread during the entire follow-up. None of these events happened during the index hospitalization and before therapeutic warfarin anticoagulation was achieved. Of all the complications, 6 needed hospital admittance.

**Table 3:** Outcome events.

	LOW-INR	CONV-INR	p-value	RR (95% CI)
Thromboembolic events	0	3	0.119	0.00 (0.00–1.45)
Hemorrhagic events	5	13	0.045	0.37 (0.14–0.98)

**Table 4:** Details of the outcome events.

	LOW-INR Group	CONVENTIONAL-INR Group
Thromboembolic events		
Symptomatic		
TIA	—	**1**
Stroke with sequelae	—	**1**
Stroke without sequelae	—	**1**
Asymptomatic	—	—
Myocardial infarction	—	—
Peripheral thrombosis	—	—
Prosthetic thrombosis	—	—
Total number of events	—	**3**
**Hemorrhagic events**		
Major	—	**3**
Non major	**5**	**10**
Fatal event	—	**2**

Three patients had a thromboembolic event, which all occurred in the CONVENTIONAL-INR group (linearized rate 3.96 per 1,000 patient- year, 95% CI 0.82–11.58) (Tables 3 and 4. Comparison of thromboembolic events between the 2 groups was not significant (P = 0.119, RR 0.00, 95% CI 0.00–1.45). The 1-sided 97.5% exact CI for the difference in primary event proportion was 0.54%, satisfying criteria for non-inferiority. There were no cases of valve thrombosis.

**Figure 1:**
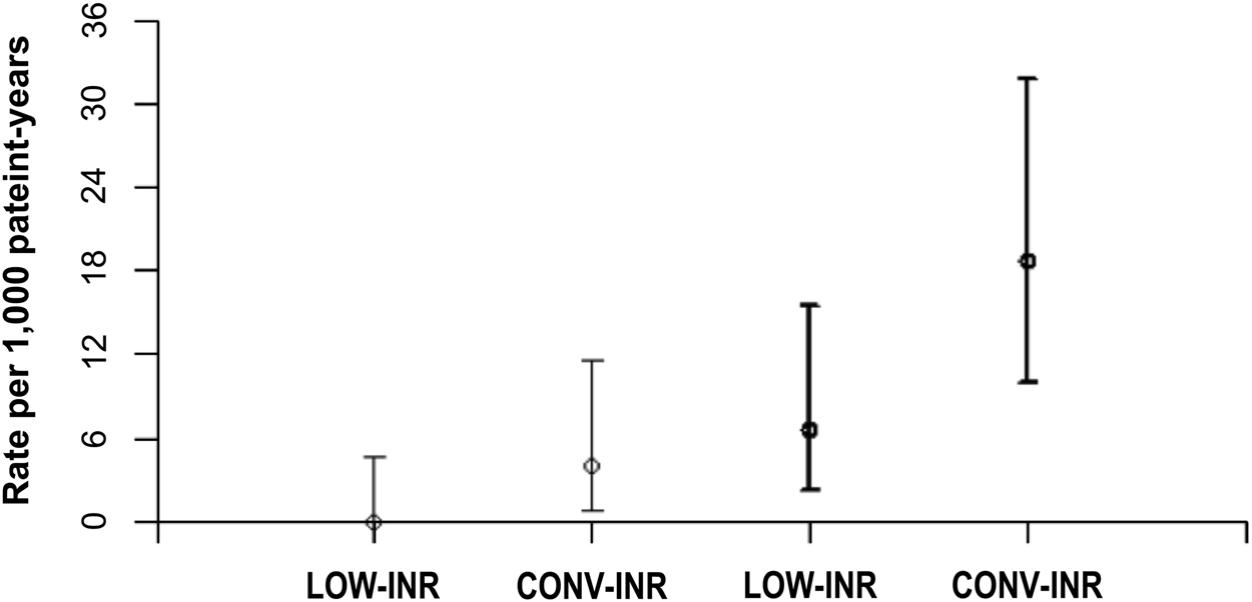
Rate per 1,000 patient-years for thromboembolic events (solid line) and for hemorrhagic events (bold solid line) for CONVENTIONAL-INR group and LOW-INR group. CONV-INR: CONVENTIONAL-INR.

The incidence of bleeding events was significantly different between the 2 groups (P = 0.045, RR 0.37, 95% CI 0.14–0.98). We observed 18 hemorrhagic events: 5 in the LOW-INR group (linearized rate 6.61 per 1,000 patient-year, 95% CI 2.14–15.41) and 13 in the CONVENTIONAL-INR group (linearized rate 18.65 per 1,000 patient-year, 95% CI 9.93–31.89). The hemorrhagic events were divided as follows: 3 major hemorrhages requiring hospitalization (in the CONVENTIONAL-INR) and 15 minor hemorrhages (10 in the CONVENTIONAL-INR and -5 in the LOW-INR group) (Tables 3 and 4). Although there were no major hemorrhages in the LOW-INR group, the significant difference in the incidence of bleeding between the 2 groups was reached by the inclusion of both major and non-major events. Linearized rate of thromboembolic and bleeding events for CONVENTIONAL-INR group and LOW-INR group are reported in Fig. 1.

Only 2 patients died: one because of a thrombotic event and the other of a cerebral hemorrhagic event. Both were in the CONVENTIONAL-INR group.

Of the 5 hemorrhagic events in the LOW-INR group, 4 occurred in patients with an INR higher of the target value, which was >3.5.

In the CONVENTIONAL-INR group, two out of the three patients experiencing a thromboembolic event had an INR within the target range. In one patient, the thromboembolic event occurred at the discontinuation of the coumarine therapy because of a procedure of tooth extraction. In this group, the 3 major hemorrhagic complications occurred in patients with an INR above the target value. In addition, in the CONVENTIONAL-INR group, 7 minor hemorrhagic events occurred in patients with an INR above the fixed target value, whereas the other 3 minor hemorrhagic events occurred in patients with an INR within the fixed target value. No case of endocarditis, atrial fibrillation, and withdrawal from the oral anticoagulant therapy occurred during follow-up.

## Discussion

The major findings of the present LOWERING-IT trial post hoc data analysis are the following: (i) in low-risk patients with isolated mechanical AVR with a LivaNova Bicarbon valve, a low anticoagulation intensity with an INR of 1.5 to 2.5 is safe and feasible; (ii) importantly, this low-intensity anticoagulation strategy is associated with a significant reduction of the average hemorrhagic events when compared to conventional therapy (INR of 2.0 to 3.0), without any increase of thromboembolic complications.

Despite the obvious limitations of VKAs protocols, there is currently no valid pharmacological alternative to their use after mechanical prosthetic heart valve replacement since novel oral non-VKA anticoagulants failed short to show improved safety and efficacy in this setting^7,8^. Current evidences show that close management of anticoagulation therapy with frequent INR monitoring significantly improves clinical outcomes^3–5^. Another route to effectively reduce VKAs complications is a better definition of anticoagulation intensity^5^. A great amount of studies has explored this strategy with conflicting results^9–12^. As disclosed by several seminal papers, while bleeding complications are closely related to anticoagulation intensity and patient features, thromboembolism is not only related to the latter risk factors interplay but also depends on individual prosthesis thrombogenicity. Indeed, subtle design and material differences imply significantly different susceptibility to thrombosis. Consequently, the more thromboresistant the individual device the lower the optimum INR range, and hence, the better the event-free patient survival. Heterogeneity in the type of prosthesis has been a major confounder in most of available studies, which have also explored different levels of reduced antioagulation. LivaNova Bicarbon bi-leaflet valve is the only one featuring a Titanium housing and selected portions of the sewing-cuff coated with a carbon layer, a unique curved leaflet design coupled with an aerofoil housing profile along with an innovative rolling hinge mechanism. Enhanced hemocompatibility, slimmer housing that maximizes the area available for a 3 blood streams (equal in amount, parallel and laminar), no-turbulent flow and total flushing of blood-exposed surfaces are the acknowledged results of such a complex engineering^13^. This prosthesis has been in clinical use since 1990 and has shown remarkable long-term results^14^. As reported by Celiento and co-workers in their 17-year follow-up study “a low incidence of embolic events was observed with a linearized rate of 0.39% ± 0.09%/patient-years after AVR”^15^. These outcomes are consistent with those disclosed by Azarnoush *et al*. in the european multicenter experience and with those forwarded by present study^16^.

To the best of our knowledge this is the first prospective randomized trial evaluating the safety and efficacy of low-dose anticoagulation after LivaNova Bicarbon aortic valve implantation in a selected surgical sample. Intriguingly, as early as 2007, Misawa and co-workers reported on their clinical experience on 44 aortic valve replacements with Bicarbon prosthesis, managed with reduced intensity anticoagulation (therapeutic INR range 1.3–1.8) coupled with anti-platelet agents (either dipyridamole or aspirin). These authors experienced a dismal rate of a thromboembolism as high as 1.16% pt-yr and hence abandoned this reduced intensity protocol^17^. Bearing in mind the obvious statistical limitations of combining and comparing different valve series, patients baseline characteristics with no selection criteria, and different INR monitoring scheduling might explain these unfavorable figures which are significantly at odds with our data^18^. Complications rates reported in our series favorably compare to those observed in the PROACT trial with the more recently released On-X valve which also displays reduced thrombogenicity^12^.

### Study Limitations

The study suffers from all obvious limitations inherent in the non-inferiority trials coupled to those derived by the post hoc data analysis design. Nevertheless, the randomized clinical trial design and the analysis of the most frequent bileaflet valve of the original study permit to calculate in this analysis accurate and unbiased estimates with only a 10% reduction in the statistical power of the original study. Outcome analysis focused on a single type of device actually enhances the clinical implication of the original study.

Finally, these data deserve to be read in a thorough clinical perspective. At first sight, study findings seem to apply mostly to low- risk patients receiving LivaNova BICARBON aortic bileaflet valve and, thus, may not be readily applicable to the overall unselect aortic mechanical valve recipients’ population and to those receiving other commercially available devices. Nevertheless, our original LOWERING-IT trial demonstrated that low-dose anticoagulation is safe and effective with different types of bileaflet prostheses. Besides, it is noteworthy that even patients with elevated risk factors for thromboembolism may be safely treated by reduced anticoagulation protocol when receiving newly developed mechanical prostheses^5^. Thus, the study add to the emerging clinical bottom line is that reduced intensity anticoagulation, under close surveillance, might be improved in larger patients subsets receiving isolated aortic valve replacement with contemporary bileaflet prostheses.

## Conclusions

Low-dose anticoagulation is safe and effective in selected patients after aortic LivaNova Bicarbon prosthesis implantation. The overall available evidence is strong enough to suggest that, given accurate patients and devices selection and matching, reduced anticoagulation intensity might be safely and effectively adopted in larger surgical subsets under close INR scheduling. Yet, larger randomized controlled trials are needed.
